# Comparative Transcriptome Analyses Reveal a Transcriptional Landscape of Human Silicosis Lungs and Provide Potential Strategies for Silicosis Treatment

**DOI:** 10.3389/fgene.2021.652901

**Published:** 2021-06-03

**Authors:** Junling Pang, Ya Luo, Dong Wei, Zhujie Cao, Xianmei Qi, Meiyue Song, Ying Liu, Zhaoguo Li, Jin Zhang, Baicun Li, Jingyu Chen, Jing Wang, Chen Wang

**Affiliations:** ^1^State Key Laboratory of Medical Molecular Biology, Department of Pathophysiology, Peking Union Medical College, Institute of Basic Medical Sciences, Chinese Academy of Medical Sciences, Beijing, China; ^2^Transplant Center, Wuxi People’s Hospital Affiliated to Nanjing Medical University, Wuxi, China; ^3^Department of Pulmonary and Critical Care Medicine/Others, Center of Respiratory Medicine, China-Japan Friendship Hospital, Beijing, China; ^4^Department of Respiratory, The Second Affiliated Hospital of Harbin Medical University, Harbin, China; ^5^Department of Thoracic Surgery and Lung Transplantation, China-Japan Friendship Hospital, Beijing, China; ^6^State Key Laboratory of Medical Molecular Biology, Department of Physiology, Peking Union Medical College, Institute of Basic Medical Sciences, Chinese Academy of Medical Sciences, Beijing, China

**Keywords:** silicosis, human cohort, transcriptomics, pathological mechanisms, candidate targets

## Abstract

Silicosis is a fatal occupational lung disease which currently has no effective clinical cure. Recent studies examining the underlying mechanism of silicosis have primarily examined experimental models, which may not perfectly reflect the nature of human silicosis progression. A comprehensive profiling of the molecular changes in human silicosis lungs is urgently needed. Here, we conducted RNA sequencing (RNA-seq) on the lung tissues of 10 silicosis patients and 7 non-diseased donors. A total of 2,605 differentially expressed genes (DEGs) and critical pathway changes were identified in human silicosis lungs. Further, the DEGs in silicosis were compared with those in idiopathic pulmonary fibrosis (IPF) and chronic obstructive pulmonary diseases (COPD), to extend current knowledge about the disease mechanisms and develop potential drugs. This analysis revealed both common and specific regulations in silicosis, along with several critical genes (e.g., *MUC5AC* and *FGF10*), which are potential drug targets for silicosis treatment. Drugs including Plerixafor and Retinoic acid were predicted as potential candidates in treating silicosis. Overall, this study provides the first transcriptomic fingerprint of human silicosis lungs. The comparative transcriptome analyses comprehensively characterize pathological regulations resulting from silicosis, and provide valuable cues for silicosis treatment.

## Introduction

Silicosis is a progressive fibrotic lung disease, which arises from the accumulation of inhaled silica particles during occupational activities (Hoy and Chambers, 2020). Increasing workers are threatening by crystalline silica due to emerging various modern industries (Barnes et al., 2019). Many countries have recently experienced outbreaks of silicosis, even in some developed countries (Rose et al., 2019). Based on the Global Burden of Disease Study, the prevalence of silicosis is about 162.4 thousand from 1990 to 2017, with 23.7 thousand new cases in the year 2017 (GBD 2017 Disease and Injury Incidence and Prevalence Collaborators, 2018). Unfortunately, current treatment for silicosis is still limited, and no effective treatments could be used to reverse its progression. For advanced silicosis, lung transplantation is one of the therapeutic options to prolong survival time (Singer et al., 2012). However, considering its several limitations, such as a short median survival rate, many contraindications, and low availability of lung donors, lung transplant is not a routine recommendation for silicosis patients (Association, 2018). Although many studies have tried to identify the pathogenesis and potential therapeutic targets of silicosis through rodent models, these results have not been translated successfully into clinical applications (Lopes-Pacheco et al., 2016; Pollard, 2016).

RNA sequencing (RNA-seq) has long been used to extend our understanding of molecular functions across a wide range of diseases (Stark et al., 2019). Several previous studies have employed RNA-seq to reveal the comprehensive molecular alterations in silicosis, using either lung tissues from animal models (Chen J. et al., 2018; Sai et al., 2019) or human cell lines (Chan et al., 2017, 2018). However, because of the genetic differences between animals and humans and the fact that the development of silicosis involves diverse cell types, a comprehensive profile of the molecular changes in the lungs of human silicosis patients is still urgently needed.

Silicosis and idiopathic pulmonary fibrosis (IPF) are both progressively fibrosing interstitial lung diseases (Wollin et al., 2019). Additionally, as a chronic inflammatory lung disease, silicosis shares some common pathogenic mechanisms with chronic obstructive pulmonary diseases (COPD) (Beamer and Shepherd, 2013). Further, epidemiological studies have shown that patients with silicosis are prone to accompany with IPF and COPD (Fazen et al., 2020; Walters, 2020). Identifying the shared and specific pathways that are modulated by the three diseases will contribute to a better understanding of the mechanisms underpinning silicosis progression. Moreover, because there are no effective drugs for treating silicosis, characterizing the mechanisms that silicosis shares with IPF or COPD may point the way to a novel use of available drugs for treating silicosis.

In this project, we performed RNA-seq analyses using lung tissues from 10 silicosis patients and 7 non-diseased donors. Through bioinformatic analysis, we aimed to reveal the key genes and pathways that participate in the progression of silicosis. In addition, we conducted transcriptomic comparisons to identify the common and unique regulations associated with silicosis, IPF (Sivakumar et al., 2019) and COPD (Morrow et al., 2017) to expand current understanding of the molecular changes in silicosis.

## Materials and Methods

A detailed description can be found in the online Supplementary Methods. The scripts used in the analysis can be found in the github repository^1^.

### Human Samples

All human lung samples were obtained from Wuxi People’s Hospital Affiliated to Nanjing Medical University. Lung tissues of silicosis patients (*n* = 10) were obtained when they received lung transplantation. Each case (transplanted lung) was sampled close to the edge of the lung, avoiding lymph nodes, large vessels, and atmospheric tubes. Lung tissues from non-diseased donors (*n* = 7) were the resected parts during lung transplantation due to size incompatibility. Though the position was not completely fixed, lymph nodes, large vessels, and atmospheric tubes were avoided during sampling. The demographic and clinical characteristics of the subjects were recorded in Supplementary Tables 1, 2. This study was approved by the Institutional Review Boards of Nanjing Medical University, and Institute of Basic Medical Sciences, Chinese Academy of Medical Sciences. All participants gave written informed consent.

### RNA Sequencing

The RNA extraction, mRNA library construction, and RNA-seq were all performed by BGI-Shenzhen, China. Briefly, the total RNA was extracted from each human lung tissues using Trizol (Invitrogen, Carlsbad, CA, United States) following the manual instruction. To purify mRNA, oligo(dT)-attached magnetic beads were used. After library construction, 100-bp paired-end reads were sequenced using BGIseq500 platform (BGI-Shenzhen, China). The raw sequencing data was filtered by removing sequencing adapters, and low-quality reads. The cleaned reads stored in FASTQ format were used in the following bioinformatics analysis.

### Bioinformatics Analysis for the RNA-Seq Data

The human reference genome (GRCh38) and the gene annotation file in ‘‘gtf’’ format were both downloaded from Ensembl release 84 version^2^. The cleaned RNA-seq reads of each sample were aligned to the human reference genome using HISAT2 (v2.1.0) software with default parameters (Kim et al., 2015; Pertea et al., 2016). Only the reads that were uniquely mapped to the genome were retained for further analysis (Supplementary Table 3). R packages GenomicFeatures (v1.38.2) and GenomicAlignments (v1.22.1) were used for calculating the read counts of genes (Lawrence et al., 2013). DEGs between silicosis patients and non-diseased donors were got using DESeq2 (v1.26.0) package (Love et al., 2014). Since there was no significant difference between silicosis patients and non-diseased donors regarding to age, sex, body mass index, and smoking status (Supplementary Table 1), we did not consider these factors in the comparison between the silicosis group and the non-diseased group. Known batch effects (see “SampleInfo.xls” in above github repository) were considered when we performed DESeq2 to identify DEGs. The cutoff to select DEGs in the diseased group was set: adjusted *p* < 0.1, and absolute fold change > 1.5 compared to non-diseased group. The R package ggplot2 (v3.3.3) was used for the generation of volcano plot for the DEGs (Wickham, 2016). Another R package pheatmap (v1.0.12)^3^ was used for the generation of heatmap for the DEGs.

### Differentially Expressed Genes in IPF or COPD

The DEGs (adjusted *p* < 0.1; absolute fold change > 1.5) in the lungs of IPF patients (*n* = 36) compared to non-diseased controls (*n* = 19) were accessed from the supplementary materials of published paper (Sivakumar et al., 2019).

The raw data of the expression profiling for lung tissues of severe COPD and controls were downloaded from NCBI GEO datasets (GSE76925) (Morrow et al., 2017). The Microarray data was background corrected, log2 transformed and quantile normalized. An empirical Bayes shrinkage method in limma package (v3.42.2) (Ritchie et al., 2015) was used to obtain the log fold change (logFC) of genes, and the *p*-values. The cutoff to select the significantly altered genes was also set to: adjusted *p* < 0.1; absolute fold change > 1.5.

### Functional Enrichment Analysis

The R package clusterProfiler (v3.14.3) was used for the Gene Ontology (GO) enrichment analysis of genes [19]. Pathway analysis was performed using MetaCore online^4^.

### Correspondence-At-Top (CAT) Curves

We used the R package “matchBox” (v1.28.0) (Irizarry et al., 2005; Ross et al., 2011) to generate the CAT curves. To compare the DEGs among diseases or datasets, we ranked genes by decreasing log fold change. Then we used “equalRank” method to compute CAT curves. The proportion of expected top ranked features was set to 0.3 when computing probability intervals. To compare the pathways between datasets, we ordered the pathways with increasing FDRs, meaning the most significant ones are at the top of the list.

### Prediction of Potential Pharmaceuticals for Silicosis

(1) To performed connectivity mapping using CMAP^5^, the symbols of DEGs were first transferred to probe IDs based on the information of Affymetrix Human Genome U133A 2.0 Array. Then the top 500 up- and down- probe sets were uploaded as signatures in CMAP. (2) The list of DEGs in silicosis were used as input in the ‘‘Drug Lookup’’ function of MetaCore (see footnote) to search the potential drugs. (3) We also searched some key genes in DrugBank database^6^ to check whether any drugs may target them.

### Quantitative Real-Time PCR and Immunohistochemical Staining

The detailed description of quantitative real-time PCR and immunohistochemical staining can be found in the online Supplementary Methods.

## Results

### Differential Gene Analysis Revealed Wide Transcriptomic Alterations in the Lungs of Silicosis Patients Compared to Non-diseased Donors

**FIGURE 1:**
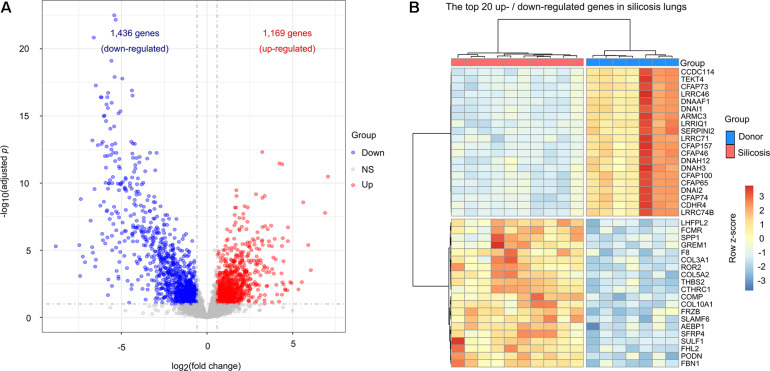
Transcriptional differences between the lungs of silicosis patients and non-diseased donors. **(A)** Volcano plot showing the significantly altered genes in silicosis lungs. Blue dots represent down-regulated genes, red dots indicate up-regulated genes, and gray dots indicate genes with no significant change. **(B)** Heatmap illustrating the top 20 most up- or down-regulated genes in the lungs of silicosis patients. Each row represents a gene, and each column a sample. The expression level of each gene was normalized using row z-score. Red indicates higher expression levels and blue indicates lower expression levels. NS, no significance.

To reveal the transcriptional changes in silicosis patients, we analyzed the gene expression in the lungs of silicosis patients (*n* = 10) versus non-diseased donors (*n* = 7). A total of 2,605 significantly altered genes between the two groups were identified, in which 1,169 genes were up-regulated and 1,436 genes were down-regulated in silicosis patients (Figure 1A and Supplementary Tables 4, 5). A heatmap of the top 20 up- and down-regulated genes (based on the adjusted *p*-value) depicts the hierarchical clusters between the two groups (Figure 1B).

Among the top 20 up-regulated genes, *COL3A1*, *COL5A2*, and *COL10A1* encode collagen proteins, which are important components of the extracellular matrix (ECM) (Saito et al., 2018). In addition, we identified 2 immune response related genes, *ROR2* and *SLAMF6*, which may play important roles in the pulmonary inflammation that accompanies silicosis (Cai et al., 2016; Wang et al., 2016). We also identified 4 genes (*SULF1*, *FHL2*, *COMP*, and *SPP1*) with profibrotic effect (Perkins et al., 2018; Posey et al., 2018; Jin et al., 2019; Morse et al., 2019). Amongst the top 20 down-regulated genes, we identified 11 genes (*CFAP73*, *CFAP46*, *CFAP157*, *CFAP74*, *CFAP65*, *CFAP100*, *CCDC114*, *TEKT4*, *ARMC3*, *DNAAF1*, and *DNAH12*) which are involved in encoding the cilia and flagella associated proteins that are essential for maintaining normal cilia function (Figure 1B; Bustamante-Marin and Ostrowski, 2017).

### Functional Enrichment Analyses Uncovered Predominant Pathway Changes in the Lungs of Silicosis Patients

**FIGURE 2:**
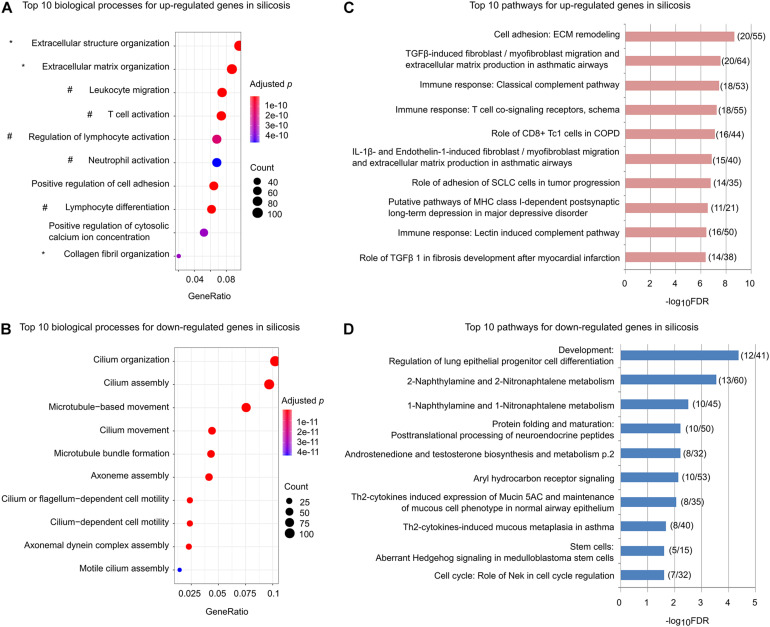
Functional enrichments for the significantly altered genes in silicosis lungs. **(A)** The top 10 biological processes for up-regulated genes. Processes related to ECM remodeling are indicated with *, while those related to immune related processes are indicated with #. The size of dots reflects the gene counts for each process. Dot color reflects adjusted *p*-values, where the most significant result is red. **(B)** The top 10 biological processes for down-regulated genes. **(C)** The top 10 significantly enriched pathways for up-regulated genes. The x-axis indicates the value of –log_1__0_FDR for each pathway. The ratios of gene count to the total genes for each pathway are shown in parentheses. **(D)** The top 10 significantly enriched pathways for the down-regulated genes.

We performed GO enrichment analysis for the up-regulated and down-regulated genes separately. As shown in Figure 2A, among the top ten significantly enriched biological processes for the up-regulated genes, three (marked by ^∗^) are related to promoting ECM remodeling and five (marked by #) are associated with regulation of lymphocyte activation, lymphocyte differentiation, and leukocyte migration (Supplementary Table 6). In contrast, the down-regulated genes were mainly associated with processes related to cilium organization, assembly, and movement (Figure 2B and Supplementary Table 7).

Similar results were found using pathway-based enrichment analysis. Pathways involving “ECM remodeling,” “TGFβ-induced fibroblast/myofibroblast migration and extracellular matrix production in asthmatic airways,” as well as immune-related pathways including “Role of CD8 + Tc1 cells in COPD,” “Classical complement pathway,” “Lectin induced complement pathway,” and “T cell co-signaling receptors, schema” were highly represented for the genes up-regulated in silicosis (Figure 2C and Supplementary Table 8). In contrast, the down-regulated genes mainly enriched in pathways including “Regulation of lung epithelial progenitor cell differentiation,” and “Th2-cytokines induced expression of Mucin 5AC and maintenance of mucous cell phenotype in normal airway epithelium” (Figure 2D and Supplementary Table 9).

Taken together, these findings indicate that mucociliary dysfunction, decreased lung epithelial progenitor cell differentiation, and enhanced profibrotic and immune-related pathways may play critical roles in silicosis progression. Critical genes in these pathways should be the focus of future investigations (Supplementary Table 10).

### Comparative Analyses Identified Common and Specific Regulations in Silicosis Lungs Compared to IPF and COPD

**FIGURE 3:**
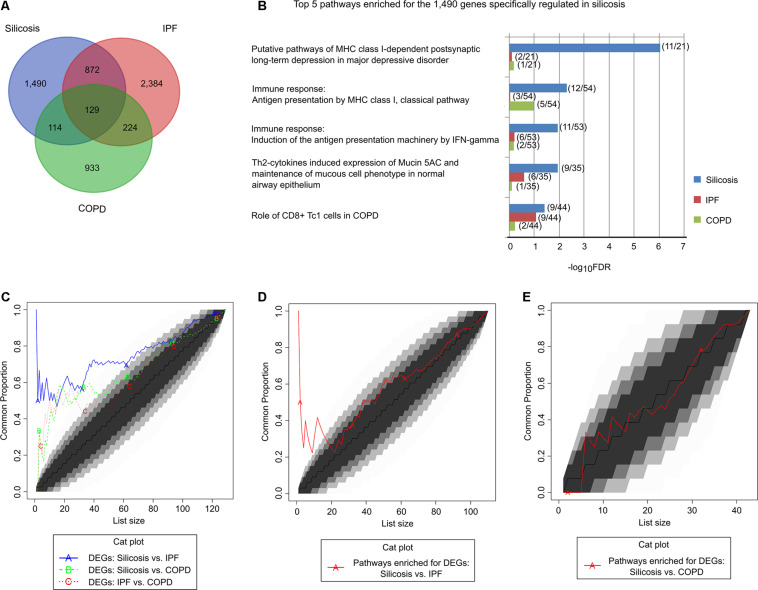
Comparison of the significantly altered genes and their pathways in silicosis, IPF, and COPD. **(A)** Venn diagram of the significantly altered genes in silicosis, IPF, and COPD. **(B)** The top 5 pathways enriched for the 1,490 genes exclusively regulated in silicosis. The enrichment results of the corresponding pathways in IPF and COPD are given for comparison. For each pathway the gene count of the differentially expressed genes compared to the total gene counts are given in parentheses. **(C)** CAT plot of the common DEGs shared among silicosis, IPF, and COPD. **(D)** CAT plot of the common significant pathways enriched for the DEGs in silicosis and IPF. **(E)** CAT plot of the common significant pathways enriched for the DEGs in silicosis and COPD.

To identify the shared and unique pathways among silicosis, IPF and COPD, we compared the DEGs in the lungs of silicosis with those previously reported for IPF (Sivakumar et al., 2019) and COPD (Morrow et al., 2017). As shown in Figure 3A, the significantly altered genes in the three lung diseases differed substantially from each other. Only 129 genes were significantly altered among all three lung diseases. In contrast, 1,490 genes (57.2%) were exclusively regulated in silicosis (Supplementary Table 11). The 129 genes that were differentially regulated in all three diseases were involved in pathways relevant for antigen presentation by MHC class II molecules, B cell signaling, T cell co-signaling receptors, SHH signaling, Hedgehog signaling and Wnt signaling (Supplementary Table 12). In contrast, the 1,490 genes specifically regulated in silicosis were mainly involved in antigen presentation by MHC class I molecules, CD8 + Tcl cells, mucociliary clearance function, epithelial progenitor cells differentiation, and OX40L/OX40 signaling (Figure 3B and Supplementary Table 13). A set of genes with canonical functions in these pathways are shown in Supplementary Table 14.

We then performed a global comparison among the three diseases using Correspondence-At-Top (CAT) curves. It was shown that the DEGs in silicosis lungs more closely resemble those got from IPF lungs than those derived from COPD (Figure 3C). The CAT curves using the ordered pathway lists for the three diseases also revealed that silicosis and IPF shares more common regulatory pathways (Figure 3D) than silicosis versus COPD (Figure 3E). In the following, we will compare silicosis with IPF and COPD separately to detect potential common or different mechanisms among diseases.

### Genes Regulated in Both Silicosis and IPF Suggest Common Profibrotic and Proinflammatory Mechanisms in Disease Development

**FIGURE 4:**
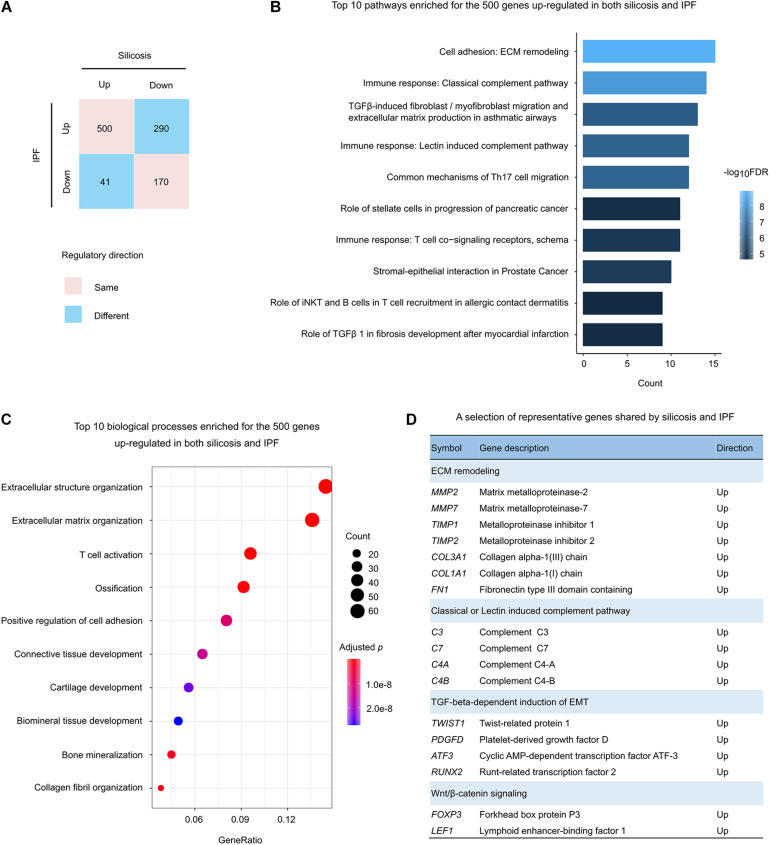
Functional analysis of the genes regulated in both silicosis and IPF. **(A)** Statistics of the gene counts regulated in both silicosis and IPF. Pink squares indicate genes regulated in the same direction (up or down) in both diseases; blue squares indicate genes regulated in different directions in the two diseases. **(B)** The top 10 pathways significantly enriched for the 500 genes up-regulated in both silicosis and IPF. **(C)** The top 10 significant biological processes enriched for the 500 genes up-regulated in both silicosis and IPF. The size of dots reflects the gene counts for each process. Dot color reflects adjusted *p*-values, where the most significant result is red. **(D)** Representative genes selected from significant pathways shared by silicosis and IPF.

There were 1,001 significantly altered genes shared between silicosis and IPF (Figure 3A). Among them, 66.9% (670 genes) were regulated in the same direction (500 up-regulated and 170 down-regulated in both diseases) (Figure 4A).

To reveal the common pathological mechanisms between silicosis and IPF, we conducted functional enrichment analysis for these genes. It was shown that the 500 up-regulated genes were largely involved in the pathways and biological processes participating in pulmonary fibrosis and immune response (Figures 4B,C and Supplementary Tables 15, 16). Relevant pathways related to pulmonary fibrosis included “ECM remodeling” and “TGFβ-induced fibroblast/myofibroblast migration and extracellular matrix production in asthmatic airways.” Immune response related pathways included “Classical complement pathway,” “Lectin induced complement pathway,” “Common mechanisms of Th17 cell migration,” and “T cell co-signaling receptors” (Figure 4B). Representative genes participating in these critical pathways are presented in Figure 4D.

Unexpectedly, the 170 down-regulated genes had no significant GO enrichment results, and only two pathways were revealed through pathway-based enrichment analysis (Supplementary Table 17). As a result, we have not discussed the function of these genes further.

### Genes Regulated in Both Silicosis and COPD Indicate Common Proinflammatory Mechanisms in Disease Development

**FIGURE 5:**
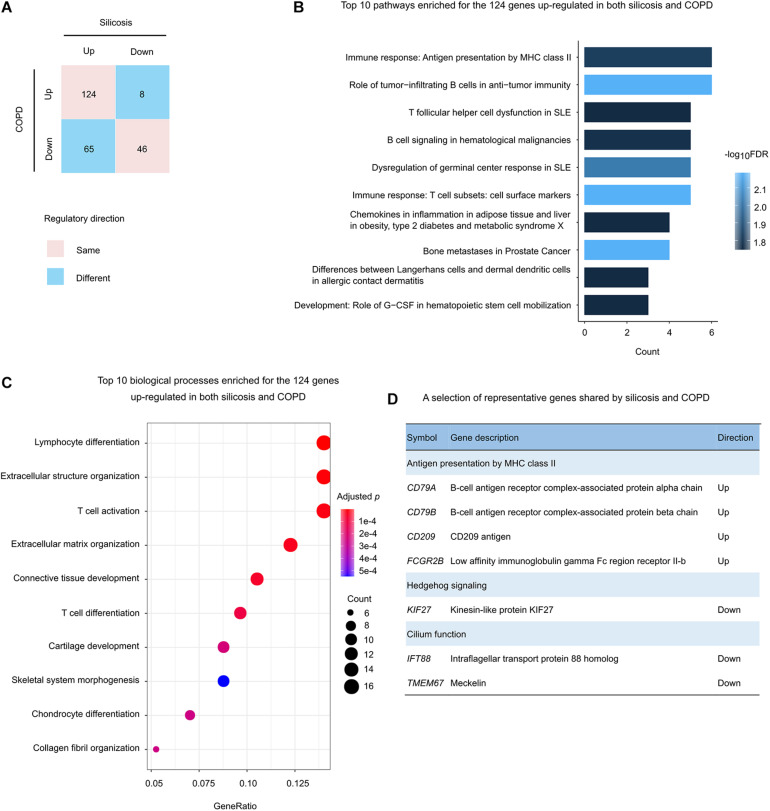
Functional analysis of the genes regulated in both silicosis and COPD. **(A)** Statistics of the gene counts regulated in both silicosis and COPD. Pink squares indicate genes regulated in the same direction (up or down) in both diseases; blue squares indicate genes regulated in different directions in the two diseases. **(B)** The top 10 significant pathways enriched for the 124 genes up-regulated in both silicosis and COPD. **(C)** The top 10 biological processes significantly enriched for the 124 genes up-regulated in both silicosis and COPD. The size of dots reflects the gene counts for each process. Dot color reflects adjusted *p*-values, where the most significant result is red. **(D)** Representative genes selected from significant pathways shared by silicosis and COPD.

There were total 243 genes that were significantly altered both in silicosis and COPD lungs (Figure 3A). About 70.0%, including 124 up-regulated and 46 down-regulated genes, were regulated in the same direction in both diseases (Figure 5A).

Pathway-based analysis showed that the 124 up-regulated genes were mainly involved in immune response pathways such as “Antigen presentation by MHC class II,” “infiltrating B cells in anti-tumor immunity,” and “T follicular helper cell dysfunction in SLE” (Figure 5B and Supplementary Table 18). Consistently, GO analysis revealed the enrichment on “Lymphocyte differentiation,” “T cell activation,” and “Extracellular structure organization” (Figures 5C,D and Supplementary Table 19).

In contrast, the 46 down-regulated genes were enriched on the pathways including “Hedgehog signaling,” “Negative regulation of Wnt/β-catenin signaling in the cytoplasm,” and “Neolacto-series GSL Metabolism” (Supplementary Table 20). In addition, the two biological processes “Cilium assembly” and “Cilium organization” were also detected through GO enrichment analysis (Figure 5D and Supplementary Table 21).

### A Summary of Key Pathways and Genes Promoting Silicosis, Experimental Validation, and Potential Drug Prediction

**FIGURE 6:**
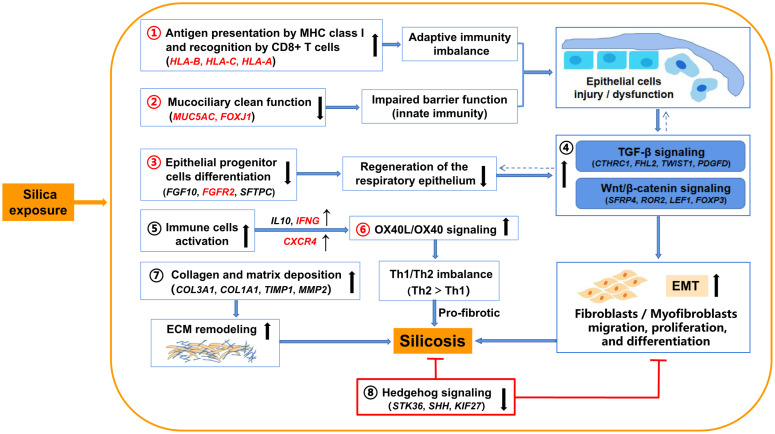
Illustration of the proposed underlying mechanisms involved in silicosis progression based on evidence from the comparative transcriptome analysis. Silica exposure promotes silicosis progression via indicated pathways (1–8). Key pathways (1–3, and 6) and genes highlighted in red are proposed to be differentially important in silicosis compared to IPF and COPD. →: promote silicosis progression; ⊤: inhibit silicosis progression; ↑: up-regulated (pathways or genes); ↓: down-regulated (pathways or genes); ←: a cross-talk between pathways.

Taken together with results from previous studies, our findings provide a comprehensive view of regulatory mechanisms in silicosis (Figure 6). Except for the common immune response pathways shared with IPF and COPD (antigen presentation by MHC class II molecules, B cell signaling, and T cell co-signaling receptors), silica exposure in silicosis may specifically drive antigen presentation by MHC class I molecules, which are recognized by CD8 + T cells, leading to damage to pulmonary epithelial cells through cytotoxic pathways, followed by pulmonary fibrosis (Zhang and Zhang, 2020). The mucociliary dysfunction combined with the weakened epithelial progenitor cells differentiation may worsen the damage of respiratory epithelium (Kotton and Morrisey, 2014; Benam et al., 2018), resulting in the up-regulation of several profibrotic pathways including TGF-β signaling and Wnt/β-catenin signaling, followed by epithelial-to-mesenchymal transition (EMT) and pulmonary fibrosis (Kage and Borok, 2012; Jones et al., 2019). Further, the activation of immune cells (e.g., Th2 cells) and their related signaling pathways such as OX40L/OX40 signaling may also cause downstream profibrotic interactions (So et al., 2006). In addition, collagen and matrix deposition promoting ECM remodeling and lung fibrosis has been widely accepted as relevant to silicosis. All these pathways (1–7 in Figure 6) eventually contribute to the inflammation and fibrosis in silicosis. In contrast, the inhibited Hedgehog signaling in silicosis (8 in Figure 6) suggests a protective feedback response to chronic epithelial injury (Zhang et al., 2019) via a mesenchymal feedback mechanism, aimed at promoting regeneration (Peng et al., 2015).

**FIGURE 7:**
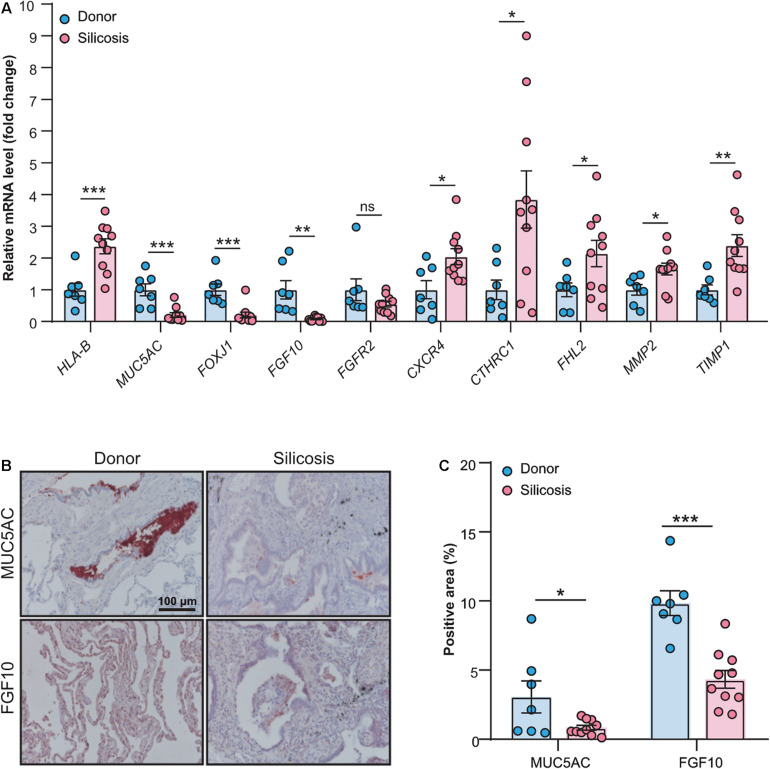
Validation of key genes revealing by RNA-seq through experimental approaches. **(A)** Relative mRNA expression levels of the genes (*HLA-B*, *MUC5AC*, *FOXJ1*, *FGF10*, *FGFR2*, *CXCR4*, *CTHRC1*, *FHL2*, *MMP2*, and *TIMP1*) in the lung tissues from silicosis patients (*n* = 10) or non-diseased donors (*n* = 7). **(B)** Representative images of immunohistochemical staining of MUC5AC and FGF10 in the lung tissues from silicosis patients or non-diseased donors. Red color indicates the positive staining. All scale bars are 100 μm. **(C)** Quantified immunoreactivity of MUC5AC and FGF10 in lung sections from silicosis patients (*n* = 10) or non-diseased donors (*n* = 7). All the quantitative results are presented as mean ± SEM. The differences were analyzed by two-tailed Student’s *t*-test. ns, no significance, *P < 0.05, **P < 0.01, ***P < 0.001.

To validate the results got from the RNA-seq data, we performed real-time quantitative PCR and immunohistochemistry (Supplementary Tables 22, 23) for some key genes that summarized in Figure 6. As shown in Figure 7A, except one gene (*FGFR2*), all other genes we tested had significantly altered mRNA levels in the lungs of patients with silicosis, the same trends as revealed by RNA-seq. Further, the protein levels of MUC5AC and FGF10 detected by immunohistochemistry were significantly decreased in silicosis lungs, especially in epithelium (Figures 7B,C). These results indicated that the genes revealed by RNA-seq could be promising therapeutic targets of silicosis.

Finally, to search the potential pharmaceuticals for silicosis, we performed connectivity mapping using CMAP to detect potential perturbagens that may reverse the biological states in silicosis (Supplementary Table 24). We also searched the potential drugs that target the key genes revealed in the RNA-seq data using MetaCore and DrugBank (Supplementary Table 25). Among the results, several drugs are of our interest. For example, Plerixafor inhibits the CXCR4 chemokine receptor, which is induced in silicosis; Retinoic acid may both inhibit the expression of COL2A1 and activate MUC5AC. Further drug tests on animal models are worthwhile to check their effects on silicosis.

## Discussion

Our study provides the first systematic exploration of the transcriptional landscape in human silicosis lungs, with the aim of identifying relevant underlying mechanisms and potential therapeutic targets for silicosis treatment. Compared to non-diseased donors, pathways involved in pro-fibrosis and immune responses were found to be significantly enhanced in silicosis lungs, and pathways related to epithelial injury and mucociliary dysfunction were also identified. The comparison between silicosis, IPF, and COPD revealed regulated pathways that were both common across diseases and specific to silicosis.

Silicosis is mainly characterized by chronic inflammation and progressive fibrosis. A growing number of studies have revealed that immune dysfunction exerts an essential role in initiating inflammation in early-stage silicosis and promoting fibrosis in the later stage. Previous studies have shown that an increase in antigen-presenting cell (APC) activity contributes to the inflammatory process. The major disease-related genes of silicosis may be mapped near the HLA-B locus (Honda et al., 1993). A recent study found an increased expression of the activation marker HLA-DR on CD8 + T cells of silica exposed workers (Brilland et al., 2019). Similarly, our transcriptomics data showed that classic APC markers of MHC class I, especially HLA-B, were specifically increased, promoting lung inflammation. The activation and infiltration of lymphocytes are reported to have important roles in silicosis. Consistently, we also observed a large number of lymphocytes infiltrated into the lungs of the advanced silicosis patients (Supplementary Table 2). Previous research demonstrated that T-helper (Th) 1 type inflammation exists in the early stage of silicosis, which is characterized by increased levels of tumor necrosis factor alpha (TNF-α) and interferon gamma (IFN-γ) (Migliaccio et al., 2008). Subsequently, the activation of Th2 cells and related signaling pathways, such as OX40L/OX40 signaling, can promote fibrotic changes (So et al., 2006). Furthermore, the regulation of C-X-C motif chemokine receptor 4/C-X-C motif chemokine ligand 12 (CXCR4/CXCL12) axis could maintain the Th2 bias, and enhance the expression of cytokines with profibrotic effect such as IL-4 and IL-10 (Piao et al., 2012; Li et al., 2020). Additionally, research on B cells in silicosis has mainly focused on IL-10 producing regulatory B cells (B10 cells). B10 cells can reduce Th1 cells and inflammation via regulating the balance of T cells, thus alleviating silica-induced lung inflammation (Liu et al., 2016; Chen et al., 2017; Lu et al., 2017). Consistently, the transcriptomic analysis of patients with advanced silicosis revealed up-regulated expression of IFN-γ, IL-10 and CXCR4, and the OX40L/OX40 signaling pathway.

Mucins and cilia play crucial roles in protecting respiratory epithelium from injury by removing inhaled particles and pathogens from the airway (Benam et al., 2018). Previous studies found that the assembly of cilia is remarkably decreased but the secretion of mucins (encoded by *MUC5AC* and *MUC5B*) is increased in COPD, asthma, IPF, and cystic fibrosis (Benam et al., 2018; Samsuzzaman et al., 2019). In contrast, our data showed that the genes and pathways associated with both the production of mucins and the assembly of cilia are significantly down-regulated in silicosis, indicating a novel feature of mucociliary dysfunction induced by silica exposure. In support of our findings, previous research has reported that *MUC5AC* deficient mice have severely impaired mucociliary clearance and acute lung injury (Koeppen et al., 2013; Roy et al., 2014). Increased secretion of MUC5AC in airway epithelial cells exhibits a protective role against influenza-induced lung injury but does not cause airway obstruction (Ehre et al., 2012). Combined with our current data, improving mucociliary function via increasing the expression of MUC5AC may be an effective way to ameliorate silicosis progression.

Lung epithelial progenitor cells play a key role in effective regeneration and repair of injured lung epithelium. Failure of lung epithelium regeneration could cause pulmonary fibrosis (Kotton and Morrisey, 2014). In our results, the pathway of lung epithelial progenitor cell differentiation, including several essential genes (e.g., *FGF10*, *FGFR2*), was remarkably down-regulated in silicosis, indicating disordered respiratory epithelium regeneration in the disease progression. Fibroblast growth factor 10 (FGF10) is essential to regulate lung epithelial regeneration after injury via binding to and activating fibroblast growth factor receptor 2 (FGFR2) on epithelial progenitors (Abler et al., 2009). FGF10-FGFR2B signaling also helps to maintain the homeostasis of alveolar type 2 (AT2) stem cells through regulating the proliferation and differentiation of lung epithelial progenitor cells (Yuan et al., 2019). Moreover, FGF10-FGFR2B signaling can help to reduce elastin and fibronectin deposition (Chen S. et al., 2018). The reduction of FGF10 expression has been observed in IPF and has been identified as a cause of IPF progression (Chanda et al., 2016). Given these previous findings and our current results, we suggest that an effective treatment for silicosis may be the promotion of epithelial cell regeneration, either through activating FGF10-FGFR2B signaling (e.g., administration of exogenous FGF10) or through stem cell-based therapy, e.g., human umbilical mesenchymal stem cells (HUMSCs) transplantation, which can effectively reduce alveolar epithelial cells injury (Li et al., 2017) and attenuate silica-induced pulmonary fibrosis (Zhang et al., 2018).

Since there are currently no effective treatments for silicosis, it is of great importance to identify potential therapeutic targets and candidate drugs. In our analyses, we adopted bioinformatic methods to predict potential drugs that may attenuate silicosis. Several drugs were identified as potential candidates for further testing in silicosis (Supplementary Tables 24, 25). Plerixafor is a small molecule drug approved for patients with non-Hodgkin’s lymphoma and multiple myeloma. Previous research has shown that plerixafor inhibits CXCR4 on CD34+ cells (Uy et al., 2008). Combined with our findings that CXCR4 was increased in silicosis lungs and the CXCR4/CXCL12 axis may contribute to the enhancement of cytokines with profibrotic effects. Thus, plerixafor may target the inflammation induced by the CXCR4/CXCL12 axis. Retinoic acid has been reported to induce the expression of airway mucins (e.g., MUC5AC) and to induce mucociliary differentiation (Yoon et al., 1997; Gray et al., 2001; Choi et al., 2004). Since the epithelial injury and mucociliary dysfunction are predominant in silicosis lungs, it is also promising to investigate the effecacy of retinoic acid in the treatment of silicosis.

As the first cohort transcriptomics study of silicosis patients, we compared our results with previous studies conducted in lung tissues of animal models or silica-induced cell lines. Genes involved in immune-related pathways or processes, including leukocytes and lymphocytes activation, were strongly enriched for significantly altered genes, consistent with findings from a silicosis mice model (Chen J. et al., 2018). We also found significant up-regulation of activating transcription factor 3 (ATF3) as in macrophages exposed to silica (Chan et al., 2018). In silica-induced A549 lung epithelial cells, immune response and inflammatory pathways are induced (Chan et al., 2017), consistent with our findings. As might be expected, there were also notable differences between our findings and previous research. The overall comparison with previous report in a silicosis mice model (Chen J. et al., 2018) using CAT curves showed that very few genes (Supplementary Figure 1A) or pathways (Supplementary Figure 1B) showed consistency with our findings. Specifically, some relevant genes revealed by previous research did not show significant alterations in our results, such as the genes *Ccl2* and *Ccr2*, which have been validated in mice silicosis models (Chen J. et al., 2018), and the DNA-binding protein inhibitor (ID) family genes, which were found to be relevant in the early responses of silicosis in epithelial cells (Chan et al., 2017). Considering differences between species and the focus of some previous research on early disease stages, our study conducted in human lungs improves our understanding of the true disease state and in particular the molecular changes underpinning silicosis.

We acknowledge limitations of our study. First, the study is limited by the small sample size. As lung transplantation is rarely conducted in silicosis patients in China, there is extremely limited availability of lung transplant tissue samples. Additionally, since the control samples were from the resected parts during lung transplantation, we were unable to decide the specific localization of sampling. The non-standardized localization of sampling from the lung may cause bias in process of comparison. To make the samples more comparable, we also sampled the silicosis lungs close to the edge of the lung. In addition, we avoided lymph nodes, large vessels, and atmospheric tubes in the sampling process. Even with these precautions, we cannot exclude the potential confounding effects caused by variability in sampling. Furthermore, the samples we used were obtained from advanced silicosis, therefore the inflammatory processes during the progression of disease may be underrepresented or underestimated. In the comparison with historical datasets (IPF and COPD), while patients in the three datasets all had advanced stage diseases and underwent lung transplantation, the different methods in sample processing and differential expression analysis may result in inaccurate conclusions, and further experimental validations are warranted. In summary, this study provides a comprehensive description of the molecular alterations in human silicosis lungs through comparative transcriptomics. The genes and pathways that characterize pathological changes in silicosis lungs may provide valuable resources and novel drug targets for silicosis.

## Data Availability Statement

The RNA-seq data of this study has been deposited in the Genome Sequence Archive (GSA) (https://bigd.big.ac.cn/gsa-human/) with the Accession number: HRA000560.

## Ethics Statement

The studies involving human participants were reviewed and approved by the Institutional Review Boards of Nanjing Medical University, and the Institute of Basic Medical Sciences, Chinese Academy of Medical Sciences. The patients/participants provided their written informed consent to participate in this study.

## Author Contributions

JC, JW, and CW designed the experiments. JW and CW obtained the funding. JC, DW, BL, and JZ collected the human lung samples. JP performed the bioinformatics analyses. JP and YLu prepared the original draft. JW, BL, ZC, XQ, MS, YLi, and ZL reviewed and edited the manuscript. All authors critically revised and approved the final manuscript.

## Conflict of Interest

The authors declare that the research was conducted in the absence of any commercial or financial relationships that could be construed as a potential conflict of interest.

## Footnotes

1 https://github.com/Pang-Junling/RNA-seq-analysis-for-human-silicosis-lungs

2 ftp://ftp.ensembl.org/pub/release-84/

3 https://CRAN.R-project.org/package=pheatmap

4 https://portal.genego.com/

5 https://portals.broadinstitute.org/cmap

6 https://go.drugbank.com/
