# Supplementary material for: Comparative Transcriptome Analyses Reveal a Transcriptional Landscape of Human Silicosis Lungs and Provide Potential Strategies for Silicosis Treatment
Source: Front Genet. 2021 Jun 3;12:652901. doi: 10.3389/fgene.2021.652901 (PMC8210851; doi:10.3389/fgene.2021.652901)
Supplement: Supplementary file 1 [file Data_Sheet_1.PDF]

The indication for transplant and human lung samples in this study

Pneumoconiosis is a rare indication for lung transplantation in China. The main indication of lung transplantation is COPD (36%), idiopathic pulmonary fibrosis (22%), cystic fibrosis (16%), and α-1-antitrypsin deficiency emphysema (7%) [1].

Silicosis patients with the following conditions can be considered for lung transplantation: resting hypoxemia; obvious anhelation after activity; obvious decrease of blood oxygen saturation; forced expiratory volume in one second (FEV1.0) < 30%; diffusion capacity for carbon monoxide of the lung (DLCO) < 40%; pulmonary hypertension; hypercapnia; cardiac function (NYHA classification) Grade Ⅲ-Ⅳ; severe changes in imaging characteristics.

All human lung samples in this study were obtained during lung transplantation from Wuxi People’s Hospital Affiliated with Nanjing Medical University. Lung tissues of silicosis patients (n = 10) were obtained when they received lung transplantation. Each case (transplanted lung) was sampled close to the edge of the lung, avoiding lymph nodes, large vessels, and atmospheric tubes. Lung tissues from non-diseased donors (n = 7) were the resected parts during lung transplantation due to size incompatibility. Though the position was not completely fixed, lymph nodes, large vessels, and atmospheric tubes were avoided during sampling.

Total RNA extraction

Total RNA was extracted from the lung tissues using Trizol (Invitrogen, Carlsbad, CA, USA) according to manual instruction. About 60 mg of tissues were ground into powder by liquid nitrogen in a 2 mL tube, followed by being homogenized for 2 minutes and rested horizontally for 5 minutes. The mix was centrifuged for 5 minutes at 12,000×g at 4℃, then the supernatant was transferred into a new EP tube with 0.3 mL chloroform/isoamyl alcohol (24:1). The mix was shacked vigorously for 15s, and then centrifuged at 12,000×g for 10 minutes at 4℃. After centrifugation, the upper aqueous phase where RNA remained was transferred into a new tube with equal volume of supernatant of isopropyl alcohol, then centrifuged at 13,600 rpm for 20 minutes at 4℃. After deserting the supernatant, the RNA pellet was washed twice with 1 mL 75% ethanol, then the mix was centrifuged at 13,600 rpm for 3 minutes at 4℃ to collect residual ethanol, followed by the pellet air dry for 5-10 minutes in the biosafety cabinet. Finally, 25μL~100μL of DEPC-treated water was added to dissolve the RNA. Subsequently, total RNA was qualified and quantified using a Nano Drop and Agilent 2100 bioanalyzer (Thermo Fisher Scientific, MA, USA).

mRNA library construction

Oligo(dT)-attached magnetic beads were used to purified mRNA. Purified mRNA was fragmented into small pieces with fragment buffer at appropriate temperature. Then First-strand cDNA was generated using random hexamer-primed reverse transcription, followed by a second-strand cDNA synthesis. afterwards, A-Tailing Mix and RNA Index Adapters were added by incubating to end repair. The cDNA fragments obtained from previous step were amplified by PCR, and products were purified by Ampure XP Beads, then dissolved in EB solution. The product was validated on the Agilent Technologies 2100 bioanalyzer for quality control. The double stranded PCR products from previous step were heated denatured and

circularized by the splint oligo sequence to get the final library. The single strand circle DNA (ssCir DNA) was formatted as the final library. The final library was amplified with phi29 to make DNA nanoball (DNB) which had more than 300 copies of one molecular, DNBs were loaded into the patterned nanoarray and pair end 100 bases reads were generated on BGIseq500 platform (BGI-Shenzhen, China).

Filtering the raw sequencing reads

The sequencing data was filtered with SOAPnuke (v1.5.2) [2] by (1) Removing reads containing sequencing adapter; (2) Removing reads whose low-quality base ratio (base quality less than or equal to 5) is more than 20%; (3) Removing reads whose unknown base ('N' base) ratio is more than 5%, afterwards clean reads were obtained and stored in FASTQ format.

Quantitative real-time PCR

Total RNA of human lungs was extracted with TRIzol regent (Invitrogen, Carlsbad, CA, USA). Then, complementary DNA (cDNA) was generated from 1 μg of total RNA using a cDNA reverse transcription kit (KR103, Tiangen Biotechnology, Beijing, China). The amplification and detection were performed using a Bio-Rad IQ5 system (Bio-Rad, Hercules, CA, USA). The SYBR Green I Q-PCR kit (TransGen Biotech, Beijing, China) was used for the amplification and the primer list is shown in the supplementary information Table S22. Gene expression, the relative level of mRNA, was calculated using the $2^{-\Delta\Delta Ct}$ method and normalized to β-actin mRNA expression level.

Immunohistochemical staining and analysis

Immunohistochemical experiment was used to evaluate the immunoreactivity of MUC5AC and FGF10 in paraffin-embedded human lung sections. Firstly, we used the mouse or rabbit anti-human antibodies (Table S23) to detect positive areas. Positive signals were visualized using goat anti-mouse or anti-rabbit secondary antibody (Table S23). Signal was developed using a 3-amino-9-ethylcarbazole reagent, and nuclei were stained using haematoxylin. Statistical pictures of immunohistochemical staining were acquired from six random microscopic fields (at 20X magnification) for each subjects. We analyzed all the above pictures by using IPWIN32 (Cybernetics Info Tech, Inc, America).

References

[1] 赵晋， 毛文君， 陆荣国， 陈员， 蒋华驰， 陈静瑜. (2018). 肺移植治疗肺尘埃沉着病的临床研究进展与问题. 器官移植. 9(5), DOI: 10.3969/j.issn.1674-7445.2018.05.013 (In Chinese)

[2] Li R, Li Y, Kristiansen K, Wang J. (2008). SOAP: short oligonucleotide alignment program. Bioinformatics. 24(5):713-4
