# Supplementary material for: Comparative Transcriptome Analyses Reveal a Transcriptional Landscape of Human Silicosis Lungs and Provide Potential Strategies for Silicosis Treatment
Source: Front Genet. 2021 Jun 3;12:652901. doi: 10.3389/fgene.2021.652901 (PMC8210851; doi:10.3389/fgene.2021.652901)
Supplement: Supplementary file 2 [file Data_Sheet_2.PDF]

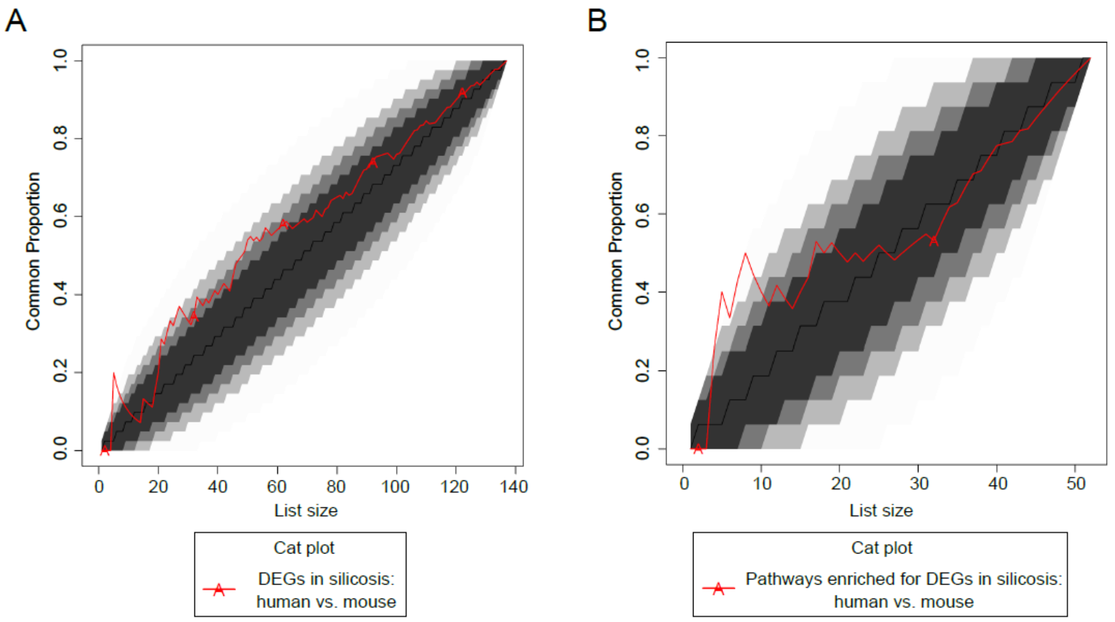

Figure S1. CAT curves to show the overall comparison between the significantly altered genes and pathways in silicosis human lungs versus silicosis mouse lungs. (A) CAT plot of the common DEGs in silicosis human lungs versus silicosis mouse lungs. (B) CAT plot of the common metacore pathways in silicosis human lungs versus silicosis mouse lungs. The DEGs in silicosis mouse lungs were got from a previous published paper (Chen et al., 2018a). Mouse genes were transferred to their corresponding human homolog (http://www.informatics.jax.org/downloads/reports/HOM_MouseHumanSequence.rpt).
